# *Leishmania* (*L.*) *mexicana* Infected Bats in Mexico: Novel Potential Reservoirs

**DOI:** 10.1371/journal.pntd.0003438

**Published:** 2015-01-28

**Authors:** Miriam Berzunza-Cruz, Ángel Rodríguez-Moreno, Gabriel Gutiérrez-Granados, Constantino González-Salazar, Christopher R. Stephens, Mircea Hidalgo-Mihart, Carlos F. Marina, Eduardo A. Rebollar-Téllez, Dulce Bailón-Martínez, Cristina Domingo Balcells, Carlos N. Ibarra-Cerdeña, Víctor Sánchez-Cordero, Ingeborg Becker

**Affiliations:** 1 Unidad de Investigación en Medicina Experimental, Facultad de Medicina, Universidad Nacional Autónoma de México, Mexico City, México; 2 Instituto de Biología, Universidad Nacional Autónoma de México, México D.F., México; 3 C3—Centro de Ciencias de la Complejidad, Universidad Nacional Autónoma de México, México D.F., México; 4 Comisión Nacional para el Conocimiento y Uso de la Biodiversidad, México D.F., 14010, México; 5 Instituto de Ciencias Nucleares, Universidad Nacional Autónoma de México, México D.F., México; 6 División Académica de Ciencias Biológicas, Universidad Juárez Autónoma de Tabasco, Villahermosa, México; 7 Centro Regional de Investigación en Salud Pública—INSP, Tapachula, Chiapas, México; 8 Laboratorio de Entomología Médica, Departamento de Zoología de Invertebrados, Facultad de Ciencias Biológicas, Universidad Autónoma de Nuevo León, San Nicolás de Los Garza, México; 9 Departamento de Ecología Humana, Centro de Investigación y de Estudios Avanzados del Instituto Politécnico Nacional (CINVESTAV), Unidad Mérida, Mérida, Yucatán, México; Lancaster University, UNITED KINGDOM

## Abstract

*Leishmania (Leishmania) mexicana* causes cutaneous leishmaniasis, an endemic zoonosis affecting a growing number of patients in the southeastern states of Mexico. Some foci are found in shade-grown cocoa and coffee plantations, or near perennial forests that provide rich breeding grounds for the sand fly vectors, but also harbor a variety of bat species that live off the abundant fruits provided by these shade-giving trees. The close proximity between sand flies and bats makes their interaction feasible, yet bats infected with *Leishmania (L.) mexicana* have not been reported. Here we analyzed 420 bats from six states of Mexico that had reported patients with leishmaniasis. Tissues of bats, including skin, heart, liver and/or spleen were screened by PCR for *Leishmania (L.) mexicana* DNA. We found that 41 bats (9.77%), belonging to 13 species, showed positive PCR results in various tissues. The infected tissues showed no evidence of macroscopic lesions. Of the infected bats, 12 species were frugivorous, insectivorous or nectarivorous, and only one species was sanguivorous (Desmodus rotundus), and most of them belonged to the family Phyllostomidae. The eco-region where most of the infected bats were caught is the Gulf Coastal Plain of Chiapas and Tabasco. Through experimental infections of two *Tadarida brasiliensis* bats in captivity, we show that this species can harbor viable, infective *Leishmania (L.) mexicana* parasites that are capable of infecting BALB/c mice. We conclude that various species of bats belonging to the family Phyllostomidae are possible reservoir hosts for *Leishmania (L.) mexicana*, if it can be shown that such bats are infective for the sand fly vector. Further studies are needed to determine how these bats become infected, how long the parasite remains viable inside these potential hosts and whether they are infective to sand flies to fully evaluate their impact on disease epidemiology.

## Author Summary

Leishmaniasis is endemic in southeastern Mexico, where *Leishmania* (*L*.) *mexicana* is the principle parasite species causing disease in humans. Previous studies using methodology for inferring potential biotic interactions between species had shown a high prevalence of bat species as potential hosts for *Leishmania*, yet bats have not been identified as hosts in Mexico. Motivated by these predictions, we currently analyze whether bats are infected with *Leishmania (L*.*) mexicana* and to what extent they serve as hosts for this parasite. We analyzed 420 bats from six states, and through PCR analysis of skin, heart, liver and/or spleen we found that 41 bats (9.77%) of 13 different bat species were infected. Most of the infected bats were netted in an eco-region characterized by plantations grown beneath shade-providing fruit trees that favors breeding conditions for the sand fly vectors and brings them in close proximity with frugivorous, insectivorous or nectarivorous bats. None of the infected bat tissues showed macroscopic lesions. Through artificial infections, we were also able to show that *Leishmania (L*.*) mexicana* remains viable and infective after passage through one species of bat. We conclude that various species of bats belonging to the family Phyllostomidae are possible reservoir hosts of *Leishmania* (*L*.) *mexicana*.

## Introduction

Many infectious diseases are zoonoses [1], where both vector and host play a crucial role in the transmission cycle of the pathogen. However, although many pathogens can have multiple hosts, there have been few systematic experimental studies conducted to identify host ranges and the relative importance of the hosts within them, as well as no systematic methods for predicting them. A systematic approach is important, as many pathogens have a potentially broad host range [2]. Additionally, the characteristics of the set of hosts of a zoonosis will play a fundamental role in determining the optimal interventions for combating the zoonosis.

Recently, a general methodology was presented [3] for inferring potential biotic interactions between species that can be used for identifying potential disease hosts and their relative importance. The method was used to predict the most important potential hosts for *Leishmania (L*.*) mexicana* in Mexico. Notable among the predictions was the high prevalence of bat species identified as important potential hosts, especially given that bats have not been previously identified as hosts in Mexico.

*Leishmania (L*.*) mexicana* is widely distributed in Mexico and the highest prevalence is found in the southeastern states of Campeche, Chiapas, Quintana Roo, Tabasco and Veracruz [4]. The disease is transmitted by the bite of phlebotomine sand flies and can infect a variety of wild and domestic mammals, whereas humans are accidental hosts [5,6]. Cutaneous leishmaniasis caused by *Leishmania (L*.*) mexicana* was first described in chicle collectors of the Yucatan peninsula in 1912 by Seidelin, hence its name “Chiclero´s Ulcer” [7]. The parasite is transmitted by various sand fly species (genus *Lutzomyia*). Known hosts include dogs [8,9] as well as various small mammals, such as *Heteromys gaumeri*, *Heteromys desmarestianus*, *Reithrodontomys gracilis*, *Marmosa mexicana*, *Sigmodon hispidus* and *Oryzomys melanotis*. Furthermore, *Ototylomys phyllotis* and *Peromyscus yucatanicus*, endemic rodents in southeast Mexico, have been incriminated as potential reservoirs based on their geographic and temporal distributions that overlap with those of vectors and patients as well as their life expectancy. The infection caused by *Leishmania (L*.*) mexicana* in these rodents is relatively benign. Although the prevalence of infected hosts can vary greatly throughout the season, the proportion of animals that become infected during their lifetime can exceed 20% [10–12].

Mexico is ranked among the top countries worldwide with the highest mammalian species richness, holding approximately 525 species, 137 of which belong to the order Chiroptera (bats). Since bat can be a potential feeding source for sand flies, it has been suspected that they might also be potential *Leishmania* hosts [13–15]. The first evidence of direct interactions between the parasite and bats were reported for *Leishmania chagasi* isolated from *Carollia perspicillata* (9.09% prevalence) [16]. Most of the previous studies on *Leishmania* spp. infections in bats revealed low prevalence: Savani *et al*. studied 659 bats belonging to 28 species in Brazil and reported 3.2% prevalence of infections with *Leishmania (L*.*) amazonensis* and *Leishmania (L*.*) infantum chagasi* (in the studies done by PCR), Mutinga reported 3% prevalence and Millán *et al*. found no evidence of *Leishmania* infections in cave bats of an endemic area in Spain. These data contrast with studies by Shapiro *et al*. that report 40% prevalence in an endemic area in Brazil [13,17,18,19].

In Mexico bats have not been studied as potential hosts for *Leishmania* infections. In this paper we present the results of an experimental study, motivated by the predictions of Stephens *et al*. (2009), to determine the extent to which bats can and do serve as hosts for *Leishmania (L*.*) mexicana* in Mexico [3].

## Methods

### Study area

The study was conducted in 22 localities including the States of Tabasco, Chiapas, Jalisco, Michoacán, Nuevo León and Veracruz. As part of a project on the dynamics of national emerging zoonosis [3], grids with cells corresponding to 25 x 25 km were analyzed. Cells were stratified according to altitudes (<2000 m or >2000 m), aiming to assign at least 80% of the cells for the analysis from the category <2000 m. The cells were also categorized into 4 types by land use and vegetation types (bodies of water, natural or modified vegetation, rural or urban settlements) and excluding cells with >50% bodies of water or urban population. Fieldwork was conducted within each 25 x 25 km in randomly selected cells.

### Bat sampling

Bats were captured using 13 mist nets of 3, 6 and 12 m, opened from 18:00 h to 24:00 h during 34 nights between February 2009 and October 2010, totaling 2,448 nights/mist-net. Mist nets were set in a diversity of habitats, including small streams, pools, along vegetation borders, vegetation clearings, cattle-rearing and crops production lands, roads and other sites where bat flying was observed. In the State of Chiapas, 276 bats were netted (65.71%), 252 of which were collected in the eco-region of the Gulf Coastal Plain, whereas 24 were netted in the mountains of the northern part of the state. In Tabasco, 104 bats (24.76%) were netted in the Gulf Coastal Plain, whereas 17 (4%) were netted in Veracruz, 16 (3.8%) in Jalisco, 2 (0.48%) in Michoacán and 5 (1.19%) in Nuevo León. Widely distributed bat species such as *Artibeus jamaicensis* and *Sturnira lilium* were netted in four states, whereas *Desmodus rotundus*, *Carollia sowelli* and *Sturnira ludovici* were only collected in three states. *Artibeus lituratus*, *Dermanura phaeotis*, *C*. *perspicillata*, *Choeroniscus godmani*, *Glossophaga soricina*, *Pteronotus parnelli*, *Pteronotus personatus* and *Myotis nigricans* are also widely distributed species, however, they were only netted in two of the collection sites and an additional 22 species were netted only in one of the six states. All captured bats were taxonomically identified to species, and sex was determined. Standard body measurements and weights were recorded. Captured bats were sacrificed according to the published guidelines and protocols of the American Society of Mammalogists [20]. All pregnant females were released at site of capture. The extracted tissues from bats (heart, liver, spleen and skin) were fixed in 90% ethanol or frozen in liquid nitrogen for PCR analysis.

### DNA purification

DNA from tissues was purified from approximately 25 mg of starting material. The DNA extractions were done with a DNeasy® Blood and Tissue kit (QIAGEN, Germany), following the manufacturer’s instructions. The genomic DNA was used for PCR-based amplification.

### Oligonucleotides

*Leishmania* infections were analyzed with two sets of primers: the first set of primers was used to detect the presence of the genus *Leishmania* and a second set of primers was used to determine the *Leishmania* species. The primers to detect *Leishmania* genus were L.MC-1S / L.MC-1R based on *Leishmania* minicircle kinetoplast DNA sequences, which is conserved among species (L.MC-1S:5´-CTRGGGGTTGGTGTAAAATAG-3´; and L.MC-1R:5´-TWTGAACGGGRTTTCTG-3´) [21]. For the identification of *Leishmania (L*.*) mexicana* species, the primer IR1, which corresponds to the 32 final nucleotides of the conserved sequences from the 3’ region of the small subunit of the 18S ribosomal gene (5’- GCT GTA GGT GAA CCT GCA GCA GCT GGA TCA TT-3’), was used as forward primer [22]. The reverse primer used was LM17 (5′-CCC CTC TCC TCCTCC CC-3′) [23].

### Polymerase chain reaction amplification

The PCR was performed using 50 μl of the following reaction mixture: *Taq* PCR Master Mix (QIAGEN, Germany) (containing a premixed solution of *Taq* DNA Polymerase, PCR buffer, MgCl_2_ and dNTPs), 100 ng of the corresponding oligonucleotides, and DNA from tissues (we used 1μl of tissue extract corresponding to 100 ng of DNA). The amplification was performed in a Perkin Elmer 2720 thermocycler using various conditions, which depended on the oligonucleotides used. *Leishmania* genus was determined with primers L.MC-1S*/* L.MC-1R and PCR amplification was performed using 30 cycles of denaturation (95°C for one minute), annealing (55°C for one minute), and polymerization (72°C for one minute). The sensitivity analysis for the primers L.MC-1S*/* L.MC-1R was made with DNA from cultured *Leishmania (L*.*) mexicana* promastigotes using: 10 ng, 1 ng, 100 pg, 10 pg, 1 pg, 100 fg, 10 fg and 1 fg DNA, and evidenced by an amplification band of 700 bp. The sensitivity for *Leishmania* DNA in tissues permitted the detection of 100 fg DNA (corresponding to 1 parasite). All the tissues that tested positive for *Leishmania* genus were additionally analyzed with primers specific for *Leishmania (L*.*) mexicana* species (with an amplification band of 790 bp), to determine whether bats were infected with the same *Leishmania (L*.*) mexicana* species as that found in patients of these endemic regions. *Leishmania (L*.*) mexicana* species was determined with primers IR1/LM17 using 35 cycles of 1 min at 94°C, 1 min at 65°C and 1 min at 72°C. In all cases, the cycles were preceded by another cycle at 94°C for 5 min and a final extension cycle of 72°C for 7 min. PCR products were analyzed by electrophoresis in 1.5% agarose gels in TAE 1X at 80 V. Gels were stained with 0.5 μg/ml ethidium bromide and photographed under a UV light source.

For additional confirmation of *Leishmania (L*.*) mexicana* infections, the amplifications obtained with primers IR1/LM17 in 2 infected bats were sequenced. For this, the PCR products were purified using the QIAquick^TM^ PCR purification kit (QIAGEN, Germany) and sequencing was done at the Molecular Biology Unit of the Instituto de Fisiología Celular, UNAM. The Automated DNA sequencing was carried out on capillary-based electrophoresis sequencers. The Unit uses an ABI Prism 310 (1-capillar) Genetic Analyzer from Applied Biosystems, with Big Dye Terminator Cycle Sequencing chemistry. The sequences were aligned with those from the National Center for Biotechnology Information, U.S. National Library of Medicine, Basic Local Alignment Search Tool (BLAST) and results confirmed that the bats were infected with *Leishmania* (*L*.) *mexicana*.

To analyze the permissiveness of bats to *Leishmania (L*.*) mexicana* infections and to ascertain whether the parasites retained their infectivity after being exposed to the immune system of the bats, we artificially infected two individuals of *Tadarida brasiliensis*, which had been netted in Metztitlan, Hidalgo and kept in captivity. These animals were infected with 1x10^6^ *Leishmania (L*.*) mexicana* promastigotes (MHOM/MX/84/SET GS, isolated from a patient with diffuse cutaneous leishmaniasis in Tabasco, Mexico) in the foot and kept in the animal facilities of the School of Medicine at UNAM, in the care of veterinary personnel according to the guidelines established by the American Society of Mammalogists [20]. After 30 days, the bats were sacrificed and fragments of the heart, liver, spleen and skin were placed in biphasic Novy-MacNeal-Nicolle (NNN) and RPMI-1640 (Gibco, Grand Island, NY) culture medium, supplemented with 10% fetal bovine serum. Cultures were analyzed daily for presence of promastigotes. Once the promastigotes appeared in the tissue cultures, they were analyzed by PCR to establish whether they were *Leishmania (L*.*) mexicana*. To confirm that these promastigotes conserved their infectivity, they were inoculated by intradermal injection into earlobes of male BALB/c mice aged to 6–8 weeks. After six weeks of infection, PCR analysis was done with the infected earlobes of BALB/c mice.

### Ethics statement

The collection of specimens was performed according to the guidelines of the American Society for the Use of Mammalogists of Wild Mammals in Research and under a collecting permit issued by the General Direction of Wildlife of Mexico (permission number SGPA/DGVS/04631/14). The infections in mice were carried out following the National Ethical Guidelines for laboratory animals NOM-062-ZOO-1999. The project was approved by the Institutional Ethics Committee of the Medical Faculty of the National Autonomous University of Mexico (UNAM) with the registration number FMED/CI/RGG/013/01/2008.

### Statistical analysis

We use a G-test to evaluate possible differences between the number of captured males and females as well as of bats that tested positive for *Leshmania (L*.*) mexicana*, assuming a hypothesis of sexual ratio of 1:1 [24]. We additionally calculated the 95% confidence intervals based on the Wilson score [25].

### Accession numbers

The gene sequences found in our study were aligned with genes reported in Genebank under the following accession numbers: *Leishmania mexicana* strain MHOM/MX/94/INDRE NBO (AF466381.1); *Leishmania mexicana* strain MHOM/MX/85/SOLIS (AJ000313.1); *Leishmania mexicana* isolate 169 clone 1 (FJ948434.1); *Leishmania mexicana* strain MHOM/MX/84/SET GS (AF466380.1); *Leishmania mexicana* strain MHOM/MX/98/UNAM RR (AF466382.1); *Leishmania mexicana* strain MHOM/GT/86/GO22 (AJ000312.1); *Leishmania mexicana* isolate 7 clone 1 (FJ948433.1); *Leishmania mexicana* strain MNYC/BZ/62/M379 (AF466383.1); *Leishmania mexicana* isolate 169 clone 4 (FJ948436.1).

## Results

We collected a total of 420 bats from 22 localities of six states, including 35 species of six families of Chiroptera. There were no statistical differences between the number of captured male (220) and female bats (200; χ^2^ = 1.38; *P* = 0.23)

### Polymerase chain reaction amplification

**Figure 1:**
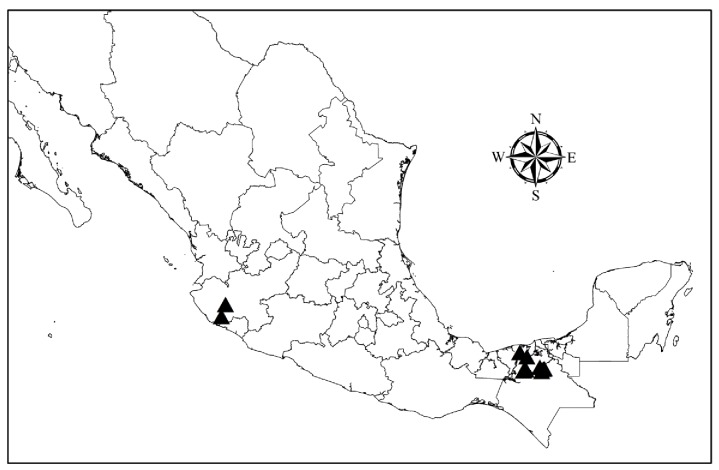
Black triangles show geographic localization of the sites in which infected bat species were collected.

The PCR analysis of tissues taken from the heart, liver, spleen and skin of the 420 bats showed that 41 (9.77%) were positive *Leishmania*. All the tissue samples that tested positive for the genus *Leishmania* also showed positive results with specific primers for *Leishmania (L*.*) mexicana* species. Of the infected bats, 25 were males and 16 females (χ^2^ = 1.97; P = 0.15), belonging to 13 species of six taxonomical families. There were no significant differences in the number of infected bats between the different families (Table 1). All the infected bats were caught in the Chiapas—Tabasco eco-region and Jalisco (Table 2, Fig. 1). 12 of the species were frugivorous, insectivorous or nectarivorous and only one species was sanguivorous (*D*. *rotundus*). There were significantly more infected bats than would be expected by random distribution in the fruit-feeding and nectar-feeding guilds (Table 1). Most of the infected bats from the eco-region of the Gulf Coastal Plain of Chiapas and Tabasco belonged to the family Phyllostomidae: *Ch*. *godmani* (23.07% infected), *Glossophaga commissarisi* (75%), *G*. *soricina* (26.92%) and *S*. *lilium* (11.11%) (Table 2). In Jalisco, only one individual of the species *Leptonycteris curasoae* and one individual of *S*. *lilium* were infected with *Leishmania* (*L*.) *mexicana*. None of the bats netted in Michoacán, Nuevo León or Veracruz were infected.

**Table 1:** 95% confidence intervals for number of species infected in different taxonomical families and trophic guilds.

Family	Num. Species	Num. Individuals	Num. Infected	Prevalence (Wilson Score)	CI (95%)
Antrozoidae	1	1	0	N/A	N/A
Emballonuridae	1	1	0	N/A	N/A
Molossidae	2	2	0	N/A	N/A
Mormoopidae	2	9	1	11	20–44
Phyllostomidae	26	402	40	10	7–13
Vespertilionidae	3	5	0	N/A	N/A
Guild					
Fruit-feeding	15	325	21	7*	4–10
Blood-feeding	2	14	1	7	1–32
Insect-feeding	14	26	8	30	6–48
Nectar-feeding	4	55	11	2*	12–32

N/A: not applicable, *Significant differences

**Table 2:** Species and family of bats netted in different Mexican states.

Family	Species	Sample size	Prevalence (%)	Confidence intervals	Chiapas	Tabasco	Jalisco	Michoacán	Nuevo León	Veracruz	Bat Gender infected
				(95%)	(+/-)	(+/-)	(+/-)	(+/-)	(+/-)	(+/-)	♂/♀
Emballonuridae	Saccopteryx bilineata	1	-	-	0/1	-	-	-	-	-	-
Molossidae	Molossus rufus	1	-	-	-	0/1	-	-	-	-	-
	Tadarida brasiliensis	1	-	-	-	-	-	-	0/1	-	-
Mormoopidae	Pteronotus personatus	4	25	5–70	1/1	0/2	-	-	-	-	1/0
	Pteronotus parnelli	5	-	-	0/4	-	0/1	-	-	-	-
	Anoura geoffroyi	6	-	-	0/6	-	-	-	-	-	-
	Artibeus jamaicensis	86	5.81	3–13	4/51	1/21	0/2	-	-	0/7	5/0
	Artibeus lituratus *	41	7.31	3–20	1/27	2/11	-	-	-	-	2/1
	Dermanura phaeotis	37	8.11	3–23	3/23	0/11	-	-	-	-	2/1
	Artibeus toltecus	1	-	-	0/1	-	-	-	-	-	-
	Dermanura watsoni	2	-	-	0/2	-	-	-	-	-	-
	Carollia perspicillata	8	-	-	0/7	0/1	-	-	-	-	-
	Carollia sowelli	45	4.44	10–23	1/34	1/7	-	-	-	0/2	0/2
	Centurio senex	1	-	-	0/1	-	-	-	-	-	-
	Chiroderma villosum	5	-	-	0/5	-	-	-	-	-	-
	Choeroniscus godmani	13	23.07	9–50	1/9	2/1	-	-	-	-	2/1
Phyllostomidae	Desmodus rotundus	14	7.14	1–33	1/6	0/1	0/6	-	-	-	1/0
	Diaemus youngi	1	-		0/1	-	-	-	-	-	-
	Glossophaga commissarisi	8	75	41–92	6/2	-	-	-	-	-	5/1
	Glossophaga soricina*	26	26.92	14–46	7/17	0/2	-	-	-	-	2/5
	Leptonycteris curasoae	2	50	-	-	-	1/1	-	-	-	1/0
	Lonchorhina aurita	1	-	-	0/1	-	-	-	-	-	-
	Micronycteris megalotis	1	-	-	0/1	-	-	-	-	-	-
	Myotis auriculus	2	-	-	-	-	-	0/2	-	-	-
	Myotis velifer	3	-	-	-	-	-	-	0/3	-	-
	Phyllostomus discolor	1	100	-	1/0	-	-	-	-	-	1/0
	Platyrrhinus helleri	5	-	-	0/5	-	-	-	-	-	
	Sturnira lilium*	63	11.11	6–21	4/20	2/28	1/0	-	-	0/8	3/4
	Sturnira ludovici	25	4.0	1–20	0/14	1/6	0/4	-	-	-	0/1
	Uroderma bilobatum	4	-	-	0/4	-	-	-	-	-	-
	Vampyrodes caraccioli	1	-	-	0/1	-	-	-	-	-	-
Antrozoidae	Antrozus pallidus	1	-	-	-	-	-	-	0/1	-	-
	Eptesicus fuscus	1	-	-	0/1	-	-	-	-	-	-
Vespertilionidae	Myotis keaysi	2	-	-	-	0/2	-	-	-	-	-
	Myotis nigricans	2	-	-	0/1	0/1	-	-	-	-	-
	Total # per state	420	-	-	30/246	9/95	2/14	0/2	0/5	0/17	25/16

List shows the numbers of *Leishmania*-infected and non-infected bats, sample size, prevalence, 95% confidence intervals as well as the percentage of infected bats according to sex.

* Species that have been reported in the literature to be infected with *Leishmania*.

**Figure 2:**
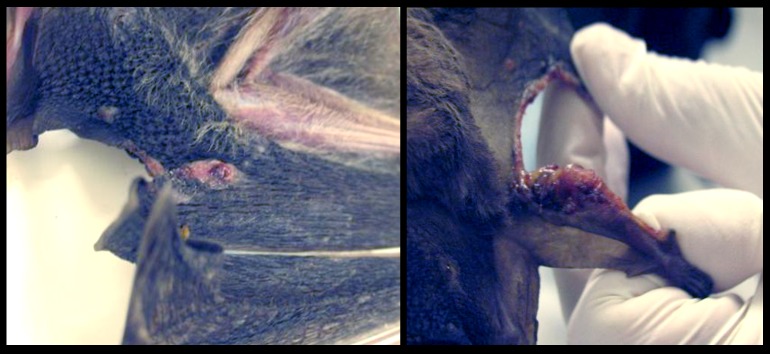
Dermal lesions in bats suggestive of *Leishmania* infection. *A*. *lituratus* found dead in Tabasco, Mexico, showing dermal lesions on the edge of the wing membrane, suggestive of a *Leishmania* infection.

None of the infected bats showed any dermal lesions and no apparent macroscopical lesions were found in the organs of the infected bats that tested positive for *Leishmania* (*L*.) *mexicana*. Only one of the bats from Tabasco (*A*. *lituratus*), which was found dead, showed dermal lesions suggestive of a *Leishmania* infection (Fig. 2). The PCR analysis of the dermal lesion of this animal was positive for *Leishmania* (*L*.) *mexicana*, and although none of the additional tissues (heart, liver, spleen) of this animal showed lesions, they tested positive for *Leishmania* (*L*.) *mexicana* by PCR.

### Permissiveness of bats to parasites

The permissiveness of bats to harbor *Leishmania (L*.*) mexicana* was shown in two *T*. *brasiliensis* individuals, which were artificially infected in captivity with 1x10^6^ *Leishmania (L*.*) mexicana* promastigotes. No visible damage was observed in the tissues, yet promastigotes were recovered from heart and liver tissue fragments of these infected bats and cultured *in vitro*. No parasites were recovered from the skin or spleen of these animals. The PCR analysis confirmed the presence of *Leishmania (L*.*) mexicana* in the infected tissues. The parasites recovered from the bat tissues were able to infect BALB/c mice, showing dermal lesions in the earlobes after six weeks of infection. PCR analysis confirmed the presence of *Leishmania (L*.*) mexicana* in the infected mice. We were thus able to prove that this bat species is able to harbor *Leishmania* (*L*.) *mexicana* parasites without showing evidence of disease, and without loss of parasite virulence, thereby rendering them potential candidates for natural *Leishmania* reservoirs.

## Discussion

In the American continent, 52 (non-flying) mammal species have been found infected by different *Leishmania* species, eight of which have been reported in Mexico [5,26–30]. Usually, there is one principal reservoir host for a given *Leishmania* species in a particular focus, whereas other mammals in the same geographic area possibly play a lesser role in disease transmission and are therefore considered minor or incidental hosts [5]. Finding the most important hosts of *Leishmania* is crucial to determine the natural transmission cycle of the parasite and to understand the epidemiology of the disease. A reservoir for *Leishmania* must ensure the subsistence and transmission of the parasite. Extensive ecological studies are needed to define a reservoir, which generally depends on the accumulation of evidence based on five criteria: (i) overlap between geographical and temporal distribution of vectors and hosts since intense host-sand fly contact is necessary; (ii) survival of the reservoir host long enough to permit transmission; (iii) infection prevalence higher than 20%, although it can vary greatly with season; (iv) parasites should be available in the skin or the blood in sufficient numbers to be infective for the sandfly vector and (v) the same *Leishmania* species that infects humans should be present in the reservoir [5,31,32]. It is noteworthy that these criteria have been questioned on the basis of the ecological impact of each reservoir host [33].

Our study showed that, according to these criteria, some of the bats fulfill at least one of the criteria to be considered potential reservoirs for *Leishmania* (*L*.) *mexicana*. In particular, a high infection rate was observed in species of Phyllostomidae, such as *Ch*. *godmani* (23.07%), *G*. *soricina* (26.92%) and *G*. *commissarisi* (75%). Additionally, seven species of Phyllostomidae showed infection rates below 12%, which suggests that these species could be considered incidental hosts (*A*. *jamaicensis*, *A*. *lituratus*, *C*. *sowelli*, *D*. *phaeotis*, *D*. *rotundus*, *S*. *lilium* and *S*. *ludovici*). Although our study found no differences in infected bat species between taxonomical families, there were more infected frugivorous and nectarivorous bats, which possibly indicates an overlapping of microhabitats while bats are feeding and roosting [34]. Interestingly, all the infected bats were netted in the eco-region of the Gulf Coastal Plain shared by Chiapas and Tabasco, a region that is considered a “hotspot” for leishmaniasis, as well as for other neglected tropical diseases [35]. Part of this region is known as “La Chontalpa” and is rich in cocoa plantations that require a shaded environment for their growth. Some of the larger trees that provide shade also produce fruits, which in turn nourish and attract bats (one such fruit is “chico zapote” that is harvested from the tree whose latex was originally used to produce chewing gum or “chicle” that gave the disease its name). It is therefore not surprising that most of the infected bat species found in this region were frugivores, insectivores, nectarivores and only one species was a sanguivore. These environmental conditions are also optimal for sand fly breeding. The humid, shaded areas, rich in organic detritus from the cocoa shells, are optimal feeding sites for *Lutzomyia* larvae. Thus, sand flies and bats co-inhabit the same nesting and feeding areas. Various *Lutzomyia* species have been found to fly up to a height of 10 m [36], which coincides with that of bats. Additionally, both bats and sand flies have their highest activity after dusk. Bats can return to the same tree up to 15 times during one night. Furthermore, sand flies can inhabit caves and crevices that are also used by bats, enabling prolonged contact between bats and sand flies, which suggests that bats may provide an alternative blood source for blood sucking female *Lutzomyia* species in such niche environments [15,37,38].

Interestingly, the “Chontalpa” area of Tabasco is also the region with the highest incidence of patients with cutaneous leishmaniasis infected with *Leishmania* (*L*.) *mexicana* [4,23,39]. Thus, this ecological niche seems optimal for *Leishmania* (*L*.) *mexicana* transmission, and where bats may play a role as potential reservoir hosts. The abundance of bats in this area, together with their longevity, is consistent with the criteria for considering the bat as a potential host, allowing the persistence and dispersal of *Leishmania (L*.*) mexicana* in this endemic area.

Many bat species exhibit differential activity depending on sex [40,41]. For instance, male bats fly less and spend more time in roosts than females. Therefore, we would expect a difference in their exposure to *Leishmania* infections, which could shed some information on mechanisms of infection in bats. Yet our study showed no statistical differences between both genders, although there was a tendency for more infected male bats. The relatively small number of infected male and female bats of our study could be the reason for not detecting any preferential infection pattern.

Bats presumably become infected via the bites of infected sand flies. Some *Lutzomyia* species, such as *Lutzomyia verspertilionis*, feed exclusively on bats and have a distinct preference for the species *C*. *perspicillata*, whereas *Lu*. *panamensis*, *Lu*. *olmeca bicolor* and *Lu*. *sanguinaria* are attracted to bats to a much lesser degree. Although some *Lutzomyia* species may become infected during blood feeding on infected bats, disease transmission to other mammals or humans is probably related to sand fly vectors that are not specifically attracted to bats [42–44]. None of the known *Leishmania* (*L*.) *mexicana* transmitting sand flies (*Lu*. *cruciata* and *Lu*. *olmeca olmeca*) of the eco-region of the Gulf Coastal Plain is known to have bat-feeding preferences. Thus, it remains to be determined how these bats become infected.

Interestingly, *Leishmania* (*L*.) *mexicana* DNA could not only be detected in the skin, but also in the heart and liver of most infected bats, due the high sensitivity of the primers LMC1S/1R that detect 100 fg DNA, corresponding to one *Leishmania* parasite. Yet none of the PCR positive tissues showed any macroscopical evidence of tissue damage. The PCR analyses also showed that the infected organs can vary between bats and that not all infected bats showed the same infected organs, which highlights the importance of analyzing different tissues in each individual.

These data seem to indicate that bats are not greatly affected by the parasites and possibly harbor *Leishmania* undamaged. The only exception was one bat (*A*. *lituratus*) that showed skin ulcers. This bat was found dead and PCR analysis proved positive for all organs tested. It is tempting to speculate that this particular animal possibly had additional diseases, making it more susceptible to the *Leishmania* parasite. Unfortunately, the advanced decomposed state of this individual did not allow further analysis regarding its possible cause of death.

These observations seem to indicate that bats are possible reservoirs of *Leishmania* (*L*.) *mexicana*, harboring the parasite without suffering disease consequences. Yet the permissiveness of *Leishmania* survival in the bat had to be determined. Since natural infection was not feasible in captivity, we therefore artificially infected two bats held in captivity for one month in order to establish if the parasite was able to survive the bat immune system. The recovery of live parasites from both bats, which could be cultured in NNN/RPMI1640 culture medium and thereafter inoculated into BALB/c mice, leading to their disease development, provided evidence that *Leishmania* (*L*.) *mexicana* remains viable and retains its infectivity after being exposed to the *T*. *brasiliensis* immune system. The recovery of viable and infectious parasites from tissues of these bats after a one-month infection, suggests the feasibility of transmission cycles involving bats.

Our study provides the first evidence that these bats are a possible natural host reservoir for *Leishmania* (*L*.) *mexicana*. The impact of this finding is that bats can provide a much greater dispersion capacity of *Leishmania* parasites, in comparison to ground dwelling rodents or the low dispersal of sand fly vectors and thus represent a greater threat for disease spread.

To our knowledge, this is the first report of the natural infection of bats with *Leishmania (L*.*) mexicana*. Previous reports on infected wildlife have focused mainly on naturally infected rodents, although wild carnivores and rabbits have been proposed as possible sylvatic reservoirs for *Leishmania infantum* [45,46,47]. So far, bats have received little attention as possible reservoirs in endemic areas (18), although they have been shown to be a blood source for some sand fly species such as *Lutzomyia longipalpis*, that is capable of feeding on at least 4 species of bats from 3 different families: *P*. *parnellii* (Mormoopidae), *Glossophaga longirostris* (Phyllostomidae), *C*. *perspicillata* (Phyllostomidae), and *Myotis oxyotus* (Vespertilionidae) [15]. We also show novel data of 10 new bat species that had not been previously shown to become infected with *Leishmania*, since infections had only been reported in three (*A*. *lituratus*, *G*. *soricina* and *S*. *lilium*) of the 13 infected bat species found in our study [17,19]. Additionally, we propose that three bat species of the family Phyllostomidae: *Ch*. *godmani*, *G*. *commissarisi* and *G*. *soricina* are potential host reservoirs of *Leishmania* (*L*.) *mexicana* in the eco-region of the Gulf Coastal Plain (Chiapas—Tabasco).

Our findings that some species of bats are naturally infected with *Leishmania* (*L*.) *mexicana* and are likely hosts to this parasite has important consequences for the geography of this disease epidemiology. Species of *Lutzomyia* sand flies are usually habitat specialists, restricting their activities at the landscape level, and having limited dispersal capabilities. As previously discussed, small non-volant mammals, as rodents and marsupials, are the only incriminated hosts of this parasite so far. These non-volant small mammals have limited dispersal capabilities, despite that some species show wide distributions. However, their activities are restricted at a landscape level. Conversely, bats have high dispersal capabilities, even though some species are habitat specialists. All species of infected bats can fly several dozens of km per night, allowing movements of this parasite at a broader geographical scale. Since almost 62% of the bat species that tested positive in our study have a wide geographic distribution and belong to different trophic guilds, a nation-wide sampling seems warranted to understand the ecology of disease transmission more thoroughly [48]. Additional studies are needed to determine how these bats become infected and how long the parasite remains viable inside these potential hosts to better evaluate their impact on the geography of disease epidemiology.
