# Correction: *Leishmania (L*.*) mexicana* Infected Bats in Mexico: Novel Potential Reservoirs

**DOI:** 10.1371/journal.pntd.0003690

**Published:** 2015-04-16

**Authors:** The PLOS Neglected Tropical Diseases Staff

Several of the values in Table 1 and Table 2 are incorrect. Please see the corrected versions of Table 1 and Table 2 here.

**Table 1:** 95% confidence intervals for number of species infected in different taxonomical families and trophic guilds.

Family	Num. Species	Num. Individuals	Num. Infected	Prevalence (%)	CI (%)
Antrozoidae	1	1	0	N/A	N/A
Emballonuridae	1	1	0	N/A	N/A
Molossidae	2	2	0	N/A	N/A
Mormoopidae	2	9	1	11	2–44
Phyllostomidae	24	397	40	10	7–13
Vespertilionidae	5	10	0	N/A	N/A
Guild					
Fruit-feeding	15	325	21	7	4–10
Blood-feeding	2	15	1	7	1–32
Insect-feeding	13	17	2	11	3–33
Nectar-feeding	5	63	17	27*	18–39

N/A: not applicable.

^*^ Higher prevalence (Odds ratio = 5.13; CI = 2.6–10.3; 95%).

**Table 2:** Species and family of bats netted in different Mexican states.

Family	Species	Sample size	Prevalence (%)	Confidence intervals	Chiapas	Tabasco	Jalisco	Michoacán	Nuevo León	Veracruz	Bat Gender infected
				(95%)	(+/-)	(+/-)	(+/-)	(+/-)	(+/-)	(+/-)	♂/♀
Emballonuridae	*Saccopteryx bilineata*	1	-	-	0/1	-	-	-	-	-	-
Molossidae	*Molossus rufus*	1	-	-	-	0/1	-	-	-	-	-
	*Tadarida brasiliensis*	1	-	-	-	-	-	-	0/1	-	-
Mormoopidae	*Pteronotus personatus*	4	25	5–70	1/1	0/2	-	-	-	-	1/0
	*Pteronotus parnelli*	5	-	-	0/4	-	0/1	-	-	-	-
Phyllostomidae	*Anoura geoffroyi*	6	-	-	0/6	-	-	-	-	-	-
	*Artibeus jamaicensis*	86	5.81	3–13	4/51	1/21	0/2	-	-	0/7	5/0
	*Artibeus lituratus* *	41	7.31	3–20	1/27	2/11	-	-	-	-	2/1
	*Dermanura phaeotis*	37	8.11	3–23	3/23	0/11	-	-	-	-	2/1
	*Artibeus toltecus*	1	-	-	0/1	-	-	-	-	-	-
	*Dermanura watsoni*	2	-	-	0/2	-	-	-	-	-	-
	*Carollia perspicillata*	8	-	-	0/7	0/1	-	-	-	-	-
	*Carollia sowelli*	45	4.44	10–23	1/34	1/7	-	-	-	0/2	0/2
	*Centurio senex*	1	-	-	0/1	-	-	-	-	-	-
	*Chiroderma villosum*	5	-	-	0/5	-	-	-	-	-	-
	*Choeroniscus godmani*	13	23.07	9–50	1/9	2/1	-	-	-	-	2/1
	*Desmodus rotundus*	14	7.14	1–33	1/6	0/1	0/6	-	-	-	1/0
	*Diaemus youngi*	1	-	-	0/1	-	-	-	-	-	-
	*Glossophaga commissarisi*	8	75	41–92	6/2	-	-	-	-	-	5/1
	*Glossophaga soricina* *	26	26.92	14–46	7/17	0/2	-	-	-	-	2/5
	*Leptonycteris curasoae*	2	50	-	-	-	1/1	-	-	-	1/0
	*Lonchorhina aurita*	1	-	-	0/1	-	-	-	-	-	-
	*Micronycteris megalotis*	1	-	-	0/1	-	-	-	-	-	-
	*Phyllostomus discolor*	1	100	-	1/0	-	-	-	-	-	1/0
	*Platyrrhinus helleri*	5	-	-	0/5	-	-	-	-	-	-
	*Sturnira lilium* *	63	11.11	6–21	4/20	2/28	1/0	-	-	0/8	3/4
	*Sturnira ludovici*	25	4.0	1–20	0/14	1/6	0/4	-	-	-	0/1
	*Uroderma bilobatum*	4	-	-	0/4	-	-	-	-	-	-
	*Vampyrodes caraccioli*	1	-	-	0/1	-	-	-	-	-	-
Antrozoidae	*Antrozous pallidus*	1	-	-	-	-	-	-	0/1	-	-
Vespertilionidae	*Eptesicus fuscus*	1	-	-	0/1	-	-	-	-	-	-
	*Myotis auriculus*	2	-	-	-	-	-	0/2	-	-	-
	*Myotis keaysi*	2	-	-	-	0/2	-	-	-	-	-
	*Myotis nigricans*	2	-	-	0/1	0/1	-	-	-	-	-
	*Myotis velifer*	3	-	-	-	-	-	-	0/3	-	-
	Total # per state	420	-	-	30/246	9/95	2/14	0/2	0/5	0/17	25/16

List shows the numbers of *Leishmania-*infected and non-infected bats, sample size, prevalence, 95% confidence intervals as well as the percentage of infected bats according to sex.

^*^ Species that have been reported in the literature to be infected with *Leishmania*.
